# Dilute and shoot approach for toxicology testing

**DOI:** 10.3389/fchem.2023.1278313

**Published:** 2023-12-11

**Authors:** Kenichi Tamama

**Affiliations:** ^1^ Clinical Laboratories, University of Pittsburgh Medical Center, Pittsburgh, PA, United States; ^2^ Department of Pathology, University of Pittsburgh School of Medicine, Pittsburgh, PA, United States; ^3^ McGowan Institute for Regenerative Medicine, University of Pittsburgh, Pittsburgh, PA, United States

**Keywords:** dilute-and-shoot, liquid chromatography-mass spectrometry, sample preparation, matrix effect, toxicology testing, urine

## Abstract

Toxicology testing is performed in clinical settings, forensic settings, and for controlling doping. Drug screening is a toxicology test to determine if drugs are present in biological samples. The most common specimen type for drug testing is urine, as drugs and/or their metabolites are often more concentrated in the urine, extending the detection window of drugs. The dilute-and-shoot method is a simple procedure used in toxicology testing, where a sample is diluted before being directly injected into the liquid chromatography-mass spectrometry (LC-MS) system. This method is easy, quick, and cost-saving, and can be used for protein-poor liquid specimens such as urine. Thus, it is reasonable and attractive for busy toxicology laboratories to combine the dilute-and-shoot method with high-resolution hyphenated-MS for urine drug screening. This method has several disadvantages, including a suboptimal detection capability for certain analytes, as well as interference from co-eluting matrix components called matrix effects, in which co-eluting matrix molecules alter the ionization efficiency of the analyte molecules at the ionization source in LC-MS, altering (mostly reducing) the analyte detection capability. The matrix effect testing is essential for the validation of LC-MS-based assays. A reasonable approach to addressing these undesirable effects would be to minimize these components. The most straightforward approach is to reduce the amounts of matrix components by using a higher dilution of the specimen and a lower volume for specimen injection. Optimization of the chromatographic separation is another reasonable approach for reducing co-eluting matrix components with the analyte.

## 1 Introduction

Toxicology testing plays a pivotal role in clinical, forensic, and doping control purposes. Clinical toxicology testing is performed to evaluate possible overdose cases and child abuse cases in the emergency department, and compliance monitoring of prescribed medicines in the pain and opioid clinics. Forensic toxicology testing is used for crime investigations related to illicit drugs and DUI assessments. Doping control testing is used to detect performance-enhancing drugs in the athletes’ biospecimens (Maurer, 1999; 2018).

Biological specimens used for toxicology testing include urine, blood (serum/plasma), saliva, hair, meconium, umbilical cord, and aqueous humor (Maurer, 2004; Liu et al., 2018; Tamama, 2021). Among them, urine is the most used specimen type for drug screening because drugs and/or their metabolites are often more concentrated, prolonging the detection window of the drugs. Urine specimens can be obtained in a non-invasive manner but not under supervision (Maurer, 2004; Liu et al., 2018; Tamama, 2021). Saliva is also utilized for toxicology testing. It can be collected on-site non-invasively under supervision (Allen et al., 2005). Blood (serum/plasma) is best suited for quantitative toxicology testing when the drug levels are correlated with the degree of intoxication (e.g., ethanol).

Drug screening is a toxicology test to evaluate the presence of drugs in biological specimens. It consists of immune-based and mass spectrometry (MS)-based testing (Liu et al., 2018; Tamama, 2021). Immune-based drug screening is conducted as an initial quick test. It is often offered as a test panel to cover the major drug classes. MS-based drug testing is performed using hyphenated-MS, which combines a mass spectrometer with a chromatograph in the instrument (Maurer, 2018; 2020; Tanna et al., 2020; Maurer, 2021). In hyphenated-MS techniques, analytes in the specimen are first separated in the chromatograph, and each chromatography-separated fraction is further interrogated by mass spectrometry. Thus, the analyte identification is made by using both retention time in the chromatogram and mass spectra of the analyte.

Sample pretreatment steps are critical for hyphenated-MS-based analysis (Wells, 2023). This is especially true for urine specimens because of the complexity of the urine matrix. The levels of the matrix components are highly variable, reflecting the body’s hydration status. The urine composition is also influenced by exposomes, such as diets and environmental contaminants/pollutants, in addition to medications (Bouatra et al., 2013; González-Domínguez et al., 2020). Extraction, enzymatic hydrolysis, and chemical derivatization are typical sample pretreatment procedures (Table 1), but they are labor-intensive and time-consuming. In contrast, the dilute-and-shoot method is an easy and simple sample pretreatment procedure, and thus it is attractive to laboratorians.

**TABLE 1:** Sample pretreatments for hyphenated mass spectrometry-based analyses.

Enzymatic hydrolysis.
• It converts conjugated metabolites (e.g., hydromorphone-3-glucuronide) into unconjugated (free) metabolites (e.g., hydromorphone) by removing hydrophilic conjugates with an enzymatic treatment (e.g., glucuronidase).
• It facilitates the downstream extraction efficiency and permits the quantitation of total metabolites (unconjugated + conjugated).
Chemical derivatization.
• It derivatizes the analytes through introduction of a specific functional group to the analytes.
• For GC-MS, it is used to mask the polarity of OH or NH groups in the analyte molecules using a derivatizing agent (e.g., bis(trimethylsilyl)-trifluoroacetamide (BSTFA) and 1% trimethylchlorosilane (TMCS)) to improve the volatility of the analytes.
Extraction.
• It separates the analytes of interest from the sample matrix.
• Liquid-liquid extraction (LLE)
o It extracts the analytes using a water-immiscible organic solvent (e.g., methylene chloride) through selective partition of the analytes into the organic solvent.
o After adding the organic solvent to the pH-adjusted specimen, the organic solvent layer is separated, evaporated, and reconstituted in another solvent.
o Non-ionized analytes with a small molecular size are best extracted by LLE.
• Solid phase extraction (SPE)
o It selectively captures the analytes with a stationary phase in the column.
o After rinsing steps, the captured analytes will be released from the column into the eluant.
o It is more selective than LLE, depending on the chemical nature of the stationary phase.
• Dilution (Dilute-and-shoot method)
o Simple specimen dilution is used *in lieu* of extraction.
o It is useable for the protein-poor liquid specimens (e.g., urine, saliva).
o It is easy, quick and cost-saving.
o It is suitable for the high-throughput testing in the busy laboratories.

The dilute-and-shoot method is a simple procedure comprised of a sample dilution before direct injection or "shooting” of the specimen into the LC-MS system. This procedure is easy, quick, and cost-saving. This simple procedure also helps increase lab productivity and thus is ideal for high-throughput testing in busy laboratories. The dilute-and-shoot method is utilized for the protein-poor liquid specimens such as urine, saliva for both targeted quantitative drug testing as well as multi-drug screening using LC-MS (Allen et al., 2005; Deventer et al., 2014; Greer et al., 2021), but it cannot be used for GC-MS, which requires the reconstitution of the extracted analytes in a volatile solvent. The most common specimen type processed with the dilute-and-shoot method in toxicology testing is urine. The dilution process is obviously non-selective, causing no analyte loss, and every analyte is retained within the specimen. Thus, the dilute-and-shoot method can allow for the most comprehensive multiclass analyte drug screening (Greer et al., 2021).

## 2 Hyphenated-MS

Among hyphenated-MS techniques, gas chromatography-mass spectrometry (GC-MS) has been regarded as a gold standard technique for drug testing (Maurer, 1992); however, analytes need to be extracted and dissolved in a volatile solvent in order to be delivered to GC in the gaseous phase (Liu et al., 2018; Tamama, 2021; Maciel et al., 2022). Thus, GC-MS cannot be used for the dilute-and-shoot approach.

Instead, analytes in aqueous conditions can be analyzed using liquid chromatography-mass spectrometry (LC-MS), which can analyze hydrophilic metabolites after phase I/II metabolism as well, even without chemical derivatization (Grebe and Singh, 2011; Peters, 2011; Tamama, 2021). Thus, LC-MS is the hyphenated-MS technique useable for the dilute-and-shoot approach. In LC-MS, a soft ionization technique, either electrospray ionization (ESI) or atmospheric pressure chemical ionization (APCI), is used to ionize the analytes with no or minimal ion fragmentation either by adduct formation (e.g., protonation) or charge separation (e.g., deprotonation), generating precursor ions (Thomson, 1998; Cech and Enke, 2001; Grebe and Singh, 2011).

**FIGURE 1:**
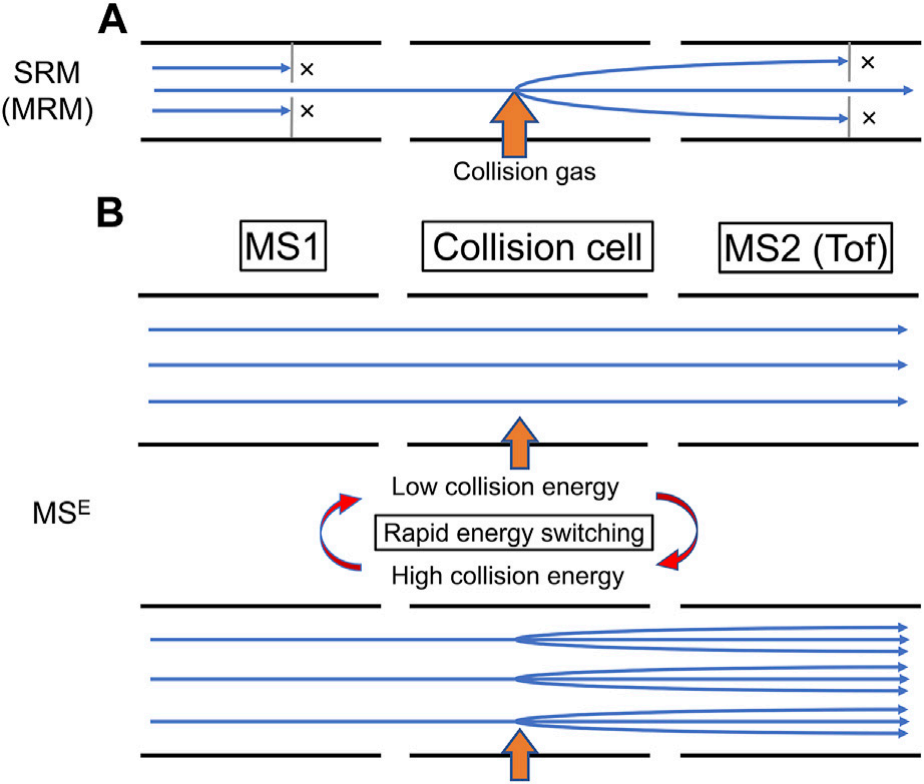
Major data acquisition modes of tandem (hybrid) quadrupole mass spectrometry. **(A)** In the single reaction monitoring (SRM)/multiple reaction monitoring (MRM) mode, a preselected ion transition is monitored. MS1 selects a precursor ion for production of product ions in the collision cell filled with the collision gas, then MS2 selects a product ion (in SRM) or product ions (in MRM). This acquisition mode is mainly used in tandem-quadrupole-MS/MS, but it is also useable in hybrid quadrupole-time of flight high-resolution mass spectrometry. **(B)** In the MS^E^ mode of hybrid quadrupole-time of flight high-resolution mass spectrometry, both low and high collision energies are applied to the collision cell via rapid energy switching, scanning both precursor ions (with low collision energy) and product ions (with high collision energy) simultaneously and non-selectively. This acquisition mode is not useable in tandem-quadrupole-MS/MS.

Tandem quadrupole-MS is the most popular MS used in LC-MS as LC-MS/MS, in which the first quadrupole can be used as a mass filter (MS1), the second quadrupole can be used as a collision cell to generate ion fragments, and the third quadrupole can be used as another mass filter (MS2) (Grebe and Singh, 2011; Zhang et al., 2016). The strength of LC-MS/MS lies in the single reaction monitoring (SRM)/multiple reaction monitoring (MRM) mode analysis, which maximizes the detection capability of the preselected target analytes by monitoring the predetermined ion transition of these analytes (Figure 1A). The SRM/MRM mode analysis can be used for drug screening of multiple preselected targets as well as quantitation of the target analytes with appropriate calibrators (Maurer, 2010).

High-resolution MS, such as time-of-flight-MS (Tof-MS) or Orbitrap-MS, is another class of MS that has gained popularity in toxicology testing. The strength of high-resolution MS lies in its capability to deduce the molecular formula of the analytes by determining the exact mass of the analytes to four decimal places and with a quadrupole-MS as MS1 in hybrid-MS to scan both precursor and product ions at the same time (e.g., MS^E^, SWATH) (Bateman et al., 2007; Gillet et al., 2012; Chindarkar et al., 2014; Whitman and Lynch, 2019; Tamama, 2021), enabling non-target data acquisition of both precursor and product ions (Figure 1B). With the retention time information for the LC-MS system used for data acquisition, this technique can theoretically identify any compounds listed in the MS library in the drug screening (Wang et al., 2019).

Both types of hyphenated-MS techniques (tandem quadrupole-MS and high-resolution MS) are used for the dilute-and-shoot method, but detection of the analytes in low levels might be challenging because the dilute-and-shoot method does not involve any analyte enrichment steps. With *a priori* knowledge of the analytes and proper SRM/MRM setting, tandem quadrupole-MS can maximize the analyte detection capabilities in the targeted analysis qualitatively and quantitatively. Thus, the combination of the dilute-and-shoot method and tandem quadrupole-MS is reasonable. On the other hand, combining the dilute-and-shoot method and non-targeted data acquisition with high-resolution MS allows for the most comprehensive drug screening because every analyte in the specimen is applied to the MS instrument. Even though multiple preselected drugs and their metabolites can be analyzed using tandem quadrupole-MS with SRM/MRM mode, the analyte coverage cannot be as comprehensive as that by non-targeted data acquisition with high-resolution MS.

## 3 Drawbacks of the dilute-and-shoot method

There are several drawbacks known for the dilute-and-shoot method. Obviously, diluted specimens contain more matrix components than post-LLE/SPE extracts; in other words, diluted specimens are dirtier than post-LLE/SPE extracts. Thus, the dilute-and-shoot method will contaminate inside the LC-MS instrument (e.g., protein buildup in the LC part and ionization source) more than post-LLE/SPE extracts, causing service disruption because of erratic test results and/or more frequent downtime, unless adequate maintenance work is given to the instrument (Wells, 2023).

Another drawback is the sub-optimal detection capability of certain analytes because it does not include any analyte enrichment steps. The levels of the analyte molecules are lowered along with those of matrix components through the dilution procedure. Thus, only the analyte molecules in high levels and/or with high ionization efficiency are analyzable by LC-MS after the dilute-and-shoot method (Kiontke et al., 2016).

Other drawbacks are related to the matrix components in the diluted specimen, which can be co-eluting, causing both false-positive and false-negative identification of the drugs and their metabolites in several mechanisms. Suppose a matrix molecule with a similar structure and molecular weight is co-eluting with the drug of interest. In that case, the matrix molecule might be misidentified as the drug by LC-MS, unless these two compounds are separated chromatographically and/or the difference in ion fragmentation patterns is evaluated. It is reasonable to assume that diluted specimens are more likely to contain isobaric matrix molecules than post-LLE/SPE extracts because matrix components are not removed through the dilution procedure. Similarly, diluted specimens are more prone to interference secondary to in-source fragmentation and matrix effects in LC-MS-based assays.

## 4 Matrix effects

Co-eluting matrix molecules are also known to alter the ionization efficiency of the analyte molecules in the soft ionization process at the ionization source of LC-MS (Bonfiglio et al., 1999; Annesley, 2003; Mei et al., 2003; Taylor, 2005; Peters and Remane, 2012; Panuwet et al., 2016). Matrix effects cause both ion suppression and enhancement, but ion suppression can diminish the peak response of the analytes and it is more problematic. Unlike the post-LLE/SLE extracts, diluted specimens contain matrix molecules in variable levels. Thus, matrix effects can be even more significant with the dilute-and-shoot method.

The major underlying mechanism of the matrix effects includes i) the competition between the analyte and co-eluted matrix component molecules for charges at the ionization source and ii) decreased formation of charged droplets due to increased surface tension and viscosity of the formed droplets, but other mechanisms are also involved in the matrix effects. (Taylor, 2005; Peters and Remane, 2012).

The matrix effect testing is essential for the validation of any LC–MS-based assays by demonstrating the consistency and reliability of any LC-MS-based assays, as suggested in the laboratory guidance (Viswanathan et al., 2007; Booth et al., 2015; Lynch, 2016). It includes the post-column infusion and post-extraction addition. In the post-column infusion, analytes of interest are continuously introduced into the LC eluent through a T-tube between LC and MS parts while a sample matrix without the analyte of interest is loaded to the LC. The effects of the matrix components on the peak response of the analytes will be monitored throughout the chromatographic run. In the post-extraction addition, the MS response of the analyte of interest in the pure solvent will be compared with its response in the sample extract (or diluted sample for the dilute-and-shoot method) (Taylor, 2005; Peters and Remane, 2012).

The matrix effect is quantitatively expressed by the matrix factor (MF), which is the ratio of analyte peak responses in the presence or absence of matrix components. The MF of 1 indicates no matrix effect in the assay, whereas the MF below 1 indicates the presence of a matrix effect in the tested specimen. An LC–MS-based assay without matrix effect is ideal, but that is not the case for diluted urine specimens. Indeed, the MF is highly variable for the diluted urine specimens because of its high variability in the matrix components and their concentrations among specimens. If available, a stable-isotope-labeled (SIL) analog of the analyte of interest can be used as the internal standard (IS) to calculate the IS normalized MF, which is the ratio of the MF of the analyte to that of SIL-IS. Because the SIL-IS exhibits similar matrix effects to matching analytes, the IS normalized MF with the SIL-IS should be close to 1 (Peters and Remane, 2012; Zhou et al., 2017).

## 5 Factors affecting the dilute-and-shoot method in toxicology testing

There are several parameters you can modify in the dilute-and-shoot procedures for test optimization.

### 5.1 Dilution factor

The matrix effects should be inversely correlated with the dilution factor. In one study, the matrix effects are minimized below 10% for 32 drugs of abuse and doping using 50- or 100-fold dilution for human urine specimens, but significant matrix effects (>20%) are still present for all drugs with a 10-fold dilution (Moreno-González et al., 2017). Higher dilutions also eliminate the matrix effects of the mouse plasma specimens. In this study, the matrix effect is minimized using a 100-fold dilution for perhexilline, but not using 50-fold or 20-fold dilutions (Esposito et al., 2016). In contrast, the dilute-and-shoot method with a 5-fold dilution of human urine specimens has significant matrix effects (−12%–87%) for the major abused drugs and their metabolites in clinical comprehensive urine drug screening (Chindarkar et al., 2014). Another study about the dilute-and-shoot method with a 10-fold dilution of human urine specimens also reveals significant matrix effects (−75%–87%) for antipsychotics and their metabolites (Feng et al., 2020). Nevertheless, the limit of quantitation (LOQ) falls between 5 and 25 ng/mL, adequately low to cover the urinary concentrations of these analytes in the patients taking these medications.

Clearly, the higher the dilution is, the less the matrix effect is. However, the dilution step also lowers the absolute concentrations of the analytes in the diluted specimen, making the analyte detection more difficult. Matrix effects for the target analytes can be monitored using SIL-analogs. Thus, the right balance between the dilution and acceptable matrix effects must be sought to attain the satisfactory limit of detection (LOD) and LOQ of the target analytes.

### 5.2 Dilution solvent

Dilution solvent is another variable of the dilute-and-shoot method. Most studies using the dilute-and-shoot method do not address the differences among various diluents. Still, the compatibilities of the diluent with the mobile phase in LC and analytes in the specimen are prerequisites.

Besides, the diluent composition can cause impact on the quality of the assay. In one study of salivary opioids using the dilute-and-shoot method, the diluent with low organic solvent (20% methanol in water) leads to better analyte separation with sharp analyte peaks. In contrast, the diluent with high organic solvent (80% methanol in water) leads to poor analyte separation with broad analyte peaks because the opioids taken from the autosampler first undergo partial desorption from the stationary phase of the reversed-phase column due to the high methanol content in the diluent, then quickly get readsorbed on the stationary phase after the adequate mixture of the diluent with surrounding aqueous mobile phase, especially with small tubing (Enders and McIntire, 2015).

### 5.3 Target analytes

Matrix effects differ tremendously among analytes in the dilute-and-shoot method. For example, the dilute-and-shoot method (5-fold dilution) of human urine specimens applied on RPLC-qTof has significant matrix effects for some drugs and metabolites (hydromorphone 57%, morphine 50%, norfentanyl (55%), noroxycodone 66%, oxymorphone 62%), but not others (buprenorphine −12%, diazepam −7%, EDDP 7%) (Chindarkar et al., 2014). Similarly, the dilute-and-shoot method with 10-fold dilution for human urine specimens applied has significant matrix effects for some drugs and metabolites (morphine 59.1%, morphine-3-glucuronide 64.3%, oxymorphone 44.2%), but not others (buprenorphine 4.9%, diazepam 8.9%, EDDP -9.9%) in another independent study (Dahlin et al., 2019).

The authors of these studies used reversed-phase LC (RPLC) with the polar mobile phase and nonpolar stationary phase (C18 group), the most common LC type used in the LC-MS-based bioassays (Hage, 2023). Polar analytes tend to elute first along with the polar unretained matrix components. Thus, polar analytes are more prone to the matrix effect in the RPLC-MS and separation of polar analytes may not be optimal for RPLC with the polar mobile phase (Müller et al., 2002). Consistently, the compounds with significant matrix effects tend to be eluting earlier than the ones with minimal matrix effects (Dahlin et al., 2019).

### 5.4 Analytical technique

Optimal chromatographic separation of the analytes and matrix components is crucial for the dilute-and-shoot method, which can be vulnerable to matrix effects. Utilization of another chromatographic technique might be helpful for certain target analytes. For example, hydrophilic interaction-liquid chromatography (HILIC)-MS, a variant of normal phase LC-MS with a polar stationary phase and an aqueous mobile phase, provides better retention and separation of polar analytes in the column (Hsieh, 2008; Cubbon et al., 2010). In the systematic evaluation of matrix effects of urine specimens for doping control, more compounds are subject to matrix effects (both ion suppression and signal enhancement) in hydrophilic interaction chromatography (HILIC) (79% of the evaluated drugs) than RPLC (36% of the evaluated drugs), because polar matrix components are better retained by HILIC than RPLC (Periat et al., 2016). But there are some compounds with matrix effects only by RPLC, but not by HILIC; for example, the matrix effects of clobenzorex and fenproporex are 91% and 98% by RPLC, but only 3% and 6% respectively by HILIC.

Another technique to minimize the matrix effects is nanospray-ESI-MS, which is used in conjunction with nanoflow-LC. Nanospray-ESI generates significantly smaller charged droplets than conventional ESI, leading to higher analyte detection capability with more tolerance to contaminated salts through more efficient ionization processes with shortened desolvation processes (Juraschek et al., 1999; Karas et al., 2000). High-resolution MS equipped with nanospray-ESI provides optimal drug detection power using the dilute-and-shoot method with 50-fold dilutions in urine drug screening. In these reports, LOQs are 5 ng/mL or lower for drugs of abuse or doping drugs with negligible matrix effects (Moreno-González et al., 2017; Alcántara-Durán et al., 2018).

## 6 Conclusion

The dilute-and-shoot approach is a very simple sample pretreatment. Unlike automatable immune-based drug screening testing, MS-based toxicology testing involves laborious sample pretreatments, prolonging the turnaround time of the tests. This is a significant impediment to the clinical toxicology situation, where clinical decision-making often hinges on the results of clinical toxicology testing. Furthermore, the caseload of clinical toxicology specimens has increased dramatically in the era of the opioid crisis and polysubstance abuse (Compton et al., 2021). There is no doubt that it appeals to busy toxicology laboratories.

Several drawbacks are known to this approach; however, these can be manageable, especially for targeted drug testing. Most of the drawbacks of the dilute-and-shoot method are attributed to the co-eluting matrix components. Thus, minimization of the co-eluting matrix components would be a reasonable approach to tackle these undesired effects caused by the co-eluting matrix components. The most straightforward approach is to minimize the amounts of matrix components by using a higher dilution of the specimen and a lower volume for specimen injection, but these measures will further lower the detection capability of the analytes. Optimization of the chromatographic separation is another reasonable approach. Laboratory personnel should be aware of the limitations of this method and use alternative methods when necessary.
